# Volumetric gray matter measures of amygdala and accumbens in childhood overweight/obesity

**DOI:** 10.1371/journal.pone.0205331

**Published:** 2018-10-18

**Authors:** Gabor Perlaki, Denes Molnar, Paul A. M. Smeets, Wolfgang Ahrens, Maike Wolters, Gabriele Eiben, Lauren Lissner, Peter Erhard, Floor van Meer, Manfred Herrmann, Jozsef Janszky, Gergely Orsi, on behalf of the I.Family Consortium

**Affiliations:** 1 MTA-PTE Clinical Neuroscience MR Research Group, Pecs, Hungary; 2 Department of Neurology, University of Pecs, Medical School, Pecs, Hungary; 3 Department of Pediatrics, University of Pecs, Medical School, Pecs, Hungary; 4 Image Sciences Institute, University Medical Center Utrecht, Utrecht University, Utrecht, Netherlands; 5 Division of Human Nutrition, Wageningen University & Research, Wageningen, Netherlands; 6 Leibniz Institute for Prevention Research and Epidemiology—BIPS, Bremen, Germany; 7 Department of Public Health and Community Medicine, University of Gothenburg, Gothenburg, Sweden; 8 Department of Biomedicine and Public Health, School of Health and Education, University of Skövde, Skövde, Sweden; 9 Center for Cognitive Sciences, University of Bremen, Bremen, Germany; 10 Department of Neuropsychology and Behavioral Neurobiology, University of Bremen, Bremen, Germany; Universidad de Jaen, SPAIN

## Abstract

**Objectives:**

Neuroimaging data suggest that pediatric overweight and obesity are associated with morphological alterations in gray matter (GM) brain structures, but previous studies using mainly voxel-based morphometry (VBM) showed inconsistent results. Here, we aimed to examine the relationship between youth obesity and the volume of predefined reward system structures using magnetic resonance (MR) volumetry. We also aimed to complement volumetry with VBM-style analysis.

**Methods:**

Fifty-one Caucasian young subjects (32 females; mean age: 13.8±1.9, range: 10.2–16.5 years) were included. Subjects were selected from a subsample of the I.Family study examined in the Hungarian center. A T_1_-weighted 1 mm^3^ isotropic resolution image was acquired. Age- and sex-standardized body mass index (zBMI) was assessed at the day of MRI and ~1.89 years (mean±SD: 689±188 days) before the examination. Obesity related GM alterations were investigated using MR volumetry in five predefined brain structures presumed to play crucial roles in body weight regulation (hippocampus, amygdala, accumbens, caudate, putamen), as well as whole-brain and regional VBM.

**Results:**

The volumes of accumbens and amygdala showed significant positive correlations with zBMI, while their GM densities were inversely related to zBMI. Voxel-based GM mass also showed significant negative correlation with zBMI when investigated in the predefined amygdala region, but this relationship was mediated by GM density.

**Conclusions:**

Overweight/obesity related morphometric brain differences already seem to be present in children/adolescents. Our work highlights the disparity between volume and VBM-derived measures and that GM mass (combination of volume and density) is not informative in the context of obesity related volumetric changes. To better characterize the association between childhood obesity and GM morphometry, a combination of volumetric segmentation and VBM methods, as well as future longitudinal studies are necessary. Our results suggest that childhood obesity is associated with enlarged structural volumes, but decreased GM density in the reward system.

## Introduction

Overweight and obese children and adolescents represent a major public health problem in many countries, including Hungary [1–3]. The rate of overweight (including obesity) among Hungarian children and adolescents aged 5–17 years is over 25% (28% and 23% for boys and girls respectively) [4]. Obesity in this age range is associated with increased risk for cardiovascular disease [5], type 2 diabetes [6] as well as other adverse conditions [7–9]. Moreover, obese youths are more likely to become obese adults [10] and as adults are more likely to develop adverse health effects [11–13].

Because the overweight/obesity in adults often originate in childhood [14, 15], it is reasonable to assume that related morphometric brain alterations are already present in children/adolescents. Moreover, in this age range brain changes may be more directly related to obesity rather than to its secondary consequences (e.g. hypertension, diabetes). Previous neuroimaging studies examining the link between obesity in children/adolescents and gray matter (GM) structure found that mainly the reward-related brain regions are affected, but the exact location and direction of differences were inconsistent: greater pallidum [16, 17], smaller thalamus and cerebellum [18], greater [19] and smaller hippocampus [17, 20, 21], decreased GM of frontal and limbic lobes (i.e. cingulum, hippocampus, parahippocampus, and amygdala) [22], greater [16] and smaller amygdala [21], smaller putamen [21], smaller caudate [21, 23] and smaller orbitofrontal cortex [24] were shown to be associated with overweight/obesity in children and adolescents.

While most of these studies assessed volumetric changes based on voxel-based morphometry (VBM), a recent study including subjects from childhood to young adulthood found that the gray matter mass (GMM; also assessed when applying VBM) does not always resemble volume [25]. More specifically, GMM is a complex combination of gray matter density (GMD) and volume, and the relative contribution of each component is unclear. Gennatas et al. (2017) found that in adolescent brain without extensive neuronal loss, GMD and volume may even change in opposite directions, therefore it is best to consider them separately, rather than mixing them into a single measure (i.e. GMM) [25]. To better characterize the association between childhood obesity and GM structural measures, studies using a combination of volumetric segmentation and VBM methods may be necessary.

Only two of the above studies used MR volumetry; one of them examined subjects with non-Caucasian origin in a narrow prepubertal age range (6–8 years) [17], while the other did not consider the possible confounding effects of head-size and examined the averaged volumes of bilateral structures [16], which may not be optimal due to hemispheric processing differences in the reward system [26, 27]. Thus, further MR volumetry studies are needed in children/adolescents to extend our knowledge regarding obesity related brain volume alterations.

Given the above neuroimaging findings and the results of our previous study showing significant positive correlation of the right amygdala volume and body mass index (BMI) in young adult males [26], we hypothesized that obesity in children/adolescents may be related to GM deviations of the reward system. Using a study design similar to our earlier study [26], we investigated the volume of five predefined reward system structures–which are presumed to play crucial roles in body weight regulation (namely the hippocampus, amygdala, accumbens, nucleus caudatus and putamen)–using MR volumetry. Contrary to our previous study in young adults, the orbitofrontal cortex was not investigated in the present study, because portions of this region were often missegmented by Freesurfer, probably due to hyperintensities around the orbitofrontal lobe [28]. The right and left structures were examined separately. Volumetric segmentation was also complemented by the VBM to contrast volume and VBM-derived measures in the context of childhood obesity related GM differences.

## Materials and methods

### Subjects

Subjects were selected from a subsample of the I.Family study examined in the Hungarian center; the study design has been described in detail elsewhere [29]. Eighty-nine healthy, Caucasian children (47 females; age range: 10–18 years) participated in the study. Our subjects watched a cartoon via MRI-compatible goggles (VisualSystem NordicNeuroLab AS, Bergen, Norway) specifically designed for fMRI to help them lie still during the structural MRI scans. This distraction strategy proved to be useful considering that none of our subjects pressed the emergency button during the MRI, however considerable movement artefacts were present in some of our subjects. After careful inspection of the images (by two independent authors: G.O. and G.P.), data of 33 subjects with visible signs of motion (e.g. blurring, ringing or ghosting) were excluded from further analyses. Later, 5 more subjects were excluded: one due to white matter abnormalities and four because the Freesurfer pipeline was unable to provide satisfactory results. Thus, 51 subjects (32 females; mean age: 13.8 ± 1.9, range: 10.2–16.5 years) were included in the final evaluation.

The puberty stages of all participants were classified based on the assessment of Tanner scale for both boys and girls and summarized in three categories: prepubertal (Tanner stage 1), peripubertal (Tanner stages 2 and 3) and pubertal (Tanner stages 4 and 5). Body weight was obtained for all subjects using a Tanita BC-418 Segmental Body Composition Analyzer (TANITA Europe GmbH, Sindelfingen, Germany), while height was measured using a Seca 225 height measuring unit (Seca Ltd., Birmingham, UK). BMI z-score (zBMI) was calculated for each subject according to the LMS method [30, 31]. This standardized overweight/obesity related measure (i.e. zBMI) was assessed at two different timepoints: ~1.89 years (mean ± SD: 689 ± 188 days) before the MRI examination (T3) and at the day of MRI (T4).

All subjects and their parents received detailed information about the investigation and parents (and children older than 12 years) gave written informed consent prior to the examination. All applicable institutional and governmental regulations concerning the ethical use of human volunteers were followed during this research. Ethical approval was obtained from the local responsible authorities in accordance with the ethical standards of the 1964 Declaration of Helsinki and its later amendments. Ethical approval was granted by the Medical Research Council–Scientific and Research Ethics Committee, Hungary 27193-2/2014/EKU (269/2014).

#### Magnetic resonance imaging

All subjects were measured on the same 3T MRI scanner (MAGNETOM Trio, Siemens AG, Erlangen, Germany) using a 12-channel head coil. An isotropic T_1_-weighted 3D MPRAGE image was acquired for each subject using a strict standardized protocol (TR/TI/TE = 2530/1100/3.37ms; Flip Angle = 7°; 176 sagittal slices; slice thickness = 1mm; FOV = 256x256mm^2^; matrix size = 256x256; receiver bandwidth = 200Hz/pixel). The protocol was based on the recommended morphometry protocols for optimal FreeSurfer reconstruction (available at: https://surfer.nmr.mgh.harvard.edu/fswiki/). Images were visually inspected in order to confirm appropriate image quality and to exclude subjects with visible brain abnormalities.

### MR volumetry

The investigated subcortical brain structures were automatically segmented by the Freesurfer 6.0 image analysis suite (https://surfer.nmr.mgh.harvard.edu). Freesurfer provides one of the most accurate automated segmentation for subcortical structures [32], especially for the hippocampus and amygdala [33, 34], both regions included in our regions of interest. Technical details were described previously [32]. Results were visually inspected by two observers (G.O. and G.P) and error correction was performed when necessary, based on the recommended troubleshooting workflow (https://surfer.nmr.mgh.harvard.edu/fswiki/FsTutorial/TroubleshootingDataV6.0). Since the volume of subcortical brain structures may be influenced by head size, total intracranial volume (ICV) estimates of Freesurfer were also extracted to allow correction in the subsequent statistical analyses.

Statistical analyses were performed using IBM SPSS Statistics for Windows, Version 20.0 (IBM Corp., Armonk, NY, USA). Multiple linear regression analyses were used to assess whether the volumes of predefined subcortical structures were associated with obesity/overweight. The volumes of subcortical structures served as dependent variables, zBMI as independent variable of interest and sex, age and ICV as additional independent variables to control for potential confounding effects. The same models were repeated replacing age by the puberty stage as a potential confounder. The assumptions of multiple linear regression were satisfied, as judged by testing for normality assumptions of the residues, outliers, independence of errors, homoscedasticity and multicollinearity [35]. Results were considered significant at two-tailed P ≤ 0.05. Uncorrected P-values are reported to facilitate comparisons to other studies. However, to account for multiple comparisons Benjamini-Hochberg correction was applied with q = 0.1 and a total number of comparisons of 20 (10 regions*2 timepoints). P-values surviving this correction are clearly marked.

#### Voxel-based morphometry

Voxel-based morphometry was performed using FSL-VBM (http://fsl.fmrib.ox.ac.uk/fsl/fslwiki/fslvbm/) [36]. First, all structural images were brain-extracted using BET. Parameter choice for this step was optimized separately for each subject and the resulting brain images were carefully reviewed to ensure appropriate brain outline estimates. Tissue-type segmentation was then carried out using FAST and the resulting GM partial volume images were affine-registered to the GM ICBM-152 standard space template and averaged together with their respective mirror images to create a first-pass, symmetric, study-specific GM template. The “original” GM partial volume images were non-linearly registered to this first-pass template and averaged together with their respective mirror images to create the final, symmetric, study-specific GM template. All “original” GM partial volume images were then non-linearly registered to the final template and concatenated into a 4D image.

The amount of GM (i.e. gray matter mass = GMM) was assessed by introducing an additional compensation (or "modulation") step for the contraction/expansion due to spatial registration, thereby correcting for volume changes due to both affine and nonlinear components of the registration (*full_modulation*) [37]. The unmodulated data were used to investigate differences in gray matter density (GMD). All images were smoothed with an isotropic Gaussian kernel (sigma = 3 mm) before statistical analyses.

Finally, a voxelwise general linear model was applied using permutation-based non-parametric testing (5000 permutations) with zBMI at T4 as variable of interest and sex and age as covariates of no interest [38]. For the GMM analysis ICV was also considered in the statistical model as a confounding variable. The same models were repeated replacing age by the puberty stage. Results were considered significant at P ≤ 0.05, corrected for multiple comparisons using “threshold-free cluster enhancement” (TFCE) [39].

Based on the observed results with volumetry, VBM analyses for GMM were also repeated using bilateral masks of the amygdala or accumbens. These masks were defined as the intersections of the original GM mask of VBM and the bilateral amygdala or accumbens labels of the Harvard-Oxford maximum probability subcortical atlas thresholded at 0%.

## Results

BMI z-score T4 and T3 were not different between sexes (P = 0.650; t = 0.456 and P = 0.953; t = -0.060 respectively, two-tailed independent samples t tests) and puberty stages (P = 0.776; t = -0.286 and P = 0.929; t = -0.090 respectively, two-tailed independent samples t tests) nor were associated with ICV (P = 0.709; r = 0.053 and P = 0.900; r = 0.018 respectively, two-tailed Pearson correlations) and age (P = 0.795; ρ = -0.037 and P = 0.830; ρ = -0.031 respectively, two-tailed Spearman correlations). Table 1 shows the demographic and obesity related characteristics of our subjects.

**Table 1:** Characteristics of study subjects (n = 51).

Sex (males/females)	19/32
Age (years)	13.8 ± 1.9 (10.2–16.5)
Puberty stage	23 peripubertal/28 pubertal
zBMI_T4	0.38 ± 1.24 (-3.15–2.80)
zBMI_T3	0.35 ± 1.25 (-3.07–3.22)
BMI classification at T4*	underweight: 3 case normal: 35 case overweight: 9 case obese: 4 case
BMI classification at T3*	underweight: 4 case normal: 38 case overweight: 5 case obese: 4 case

zBMI = BMI z-score; T4 = timepoint of MRI examination; T3 = 689 ± 188 days before the day of MRI

Values are given as mean ± standard deviation (range).

*BMI classification was established according to Cole et al.[40]

### MR volumetry

After controlling for the confounding effects of age, sex and ICV, the left and right amygdala volumes were positively associated with zBMI assessed either at the time of MRI (T4) or ~1.89 years earlier (T3). Left and right accumbens volumes showed significant positive relationships with zBMI at T4, while using zBMI at T3 only the right accumbens showed significant association (Table 2).

**Table 2:** Association of investigated brain structure volumes with zBMI.

	zBMI T4		zBMI T3	
	P-value	t-value	P-value	t-value
Right-sided structures:				
Hippocampus	0.681	-0.414	0.690	0.402
Amygdala	**0.010**^a^	**2.706**^a^	**0.008**^a^	**2.781**^a^
Accumbens region	**0.002**	**3.288**	**0.023**	**2.350**
Nucleus caudatus	0.549	-0.604	0.210	-1.272
Putamen	0.458	0.748	0.572	0.569
Left-sided structures:				
Hippocampus	0.948	-0.066	0.414	0.825
Amygdala	**0.010**^a^	**2.691**^a^	**0.002**^a^	**3.204**^a^
Accumbens region	**0.032**	**2.213**	0.169	1.398
Nucleus caudatus	0.720	0.360	0.782	-0.279
Putamen	0.269	1.120	0.353	0.938

zBMI = BMI z-score; T4 = timepoint of MRI examination; T3 = 689 ± 188 days before the day of MRI

P-values and t-values are specific to zBMI-related term in the multiple linear regression analyses adjusted for age, intracranial volume and sex.

^a^In these models the significant age*ICV interaction term was also included.

The uncorrected P-values in bold survive Benjamini–Hochberg correction for multiple comparisons calculated using q = 0.1 and a total number of comparisons of 20 (10 regions*2 timepoints).

There were no significant interactive effects of age*zBMI, sex*ICV, sex*zBMI, ICV*zBMI on the regional brain volumes of accumbens and amygdala. In cases where significant age*ICV interaction was detected (i.e. left and right amygdala), the interaction term was also included. These cases are clearly marked in Table 2, which shows the statistical results for all pre-defined brain structures. For the left amygdala significant age*sex interaction was also detected. However, after including both age*ICV and age*sex in the same model, only the age*ICV remained significant suggesting that age*sex interaction was driven by sexual dimorphism in head size.

Replacing age by the puberty stage did not change the significance pattern of our results.

#### Voxel-based morphometry

**Fig 1:**
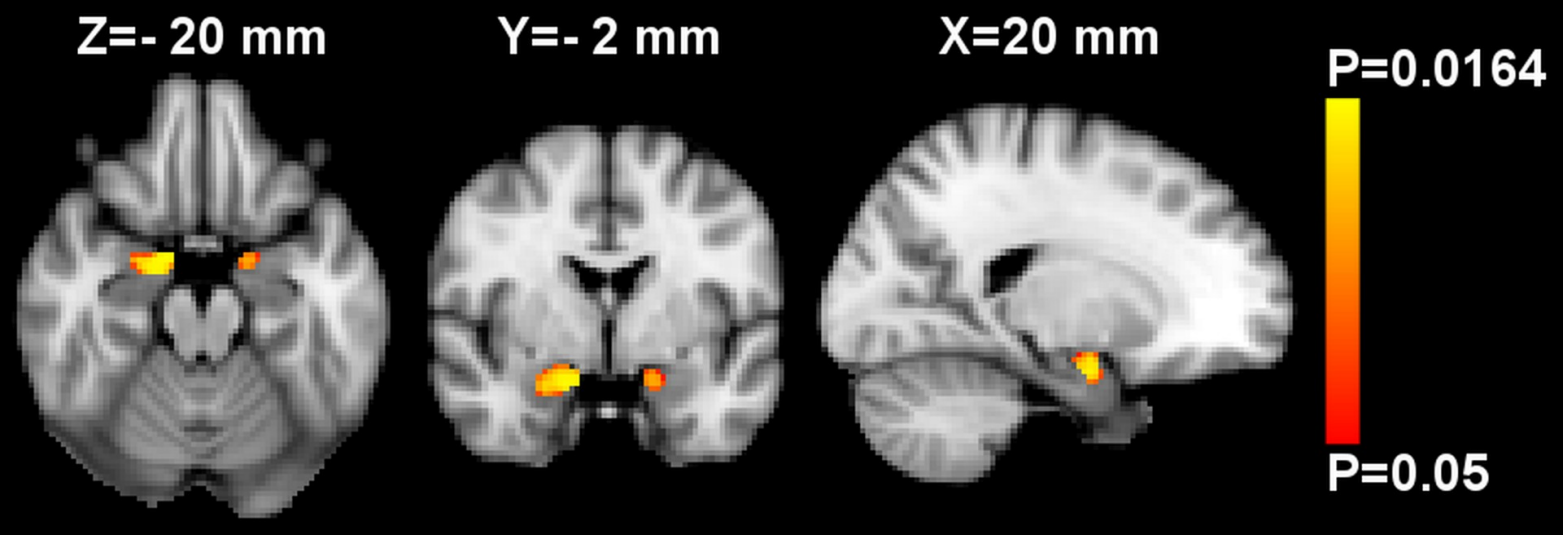
Region of interest voxel-based morphometry analysis of gray matter mass (GMM) in the bilateral mask of amygdala. Red-yellow shows voxels demonstrating significant inverse association between GMM and BMI z-score at T4 (i.e. at the day of MRI) after controlling for age, sex and intracranial volume. Color bar represents P-values corrected for multiple comparisons using “threshold-free cluster enhancement”. The map of P-values was thresholded using corrected P≤0.05. The background image is the MNI152 standard space T_1_ template. X-, Y- and Z-values indicate the MNI slice coordinates in millimeter. Images are shown in radiological convention.

Using whole-brain VBM analysis controlled for age, sex and ICV, no significant associations between GMM and zBMI were observed. Performing region of interest VBM analyses in the amygdala and accumbens, a significant inverse relationship between zBMI and GMM was found bilaterally in the amygdala (Fig 1), while the analysis of nucleus accumbens showed no significant results. However, the mean GMM extracted from the significant amygdalar voxels was no further associated with zBMI when controlling for mean GMD by partial correlation (S1 Fig; two-tailed P = 0.246, r = -0.167 and P = 0.598, r = -0.076 for the left and right sides respectively, two-tailed partial correlation analyses).

**Fig 2:**
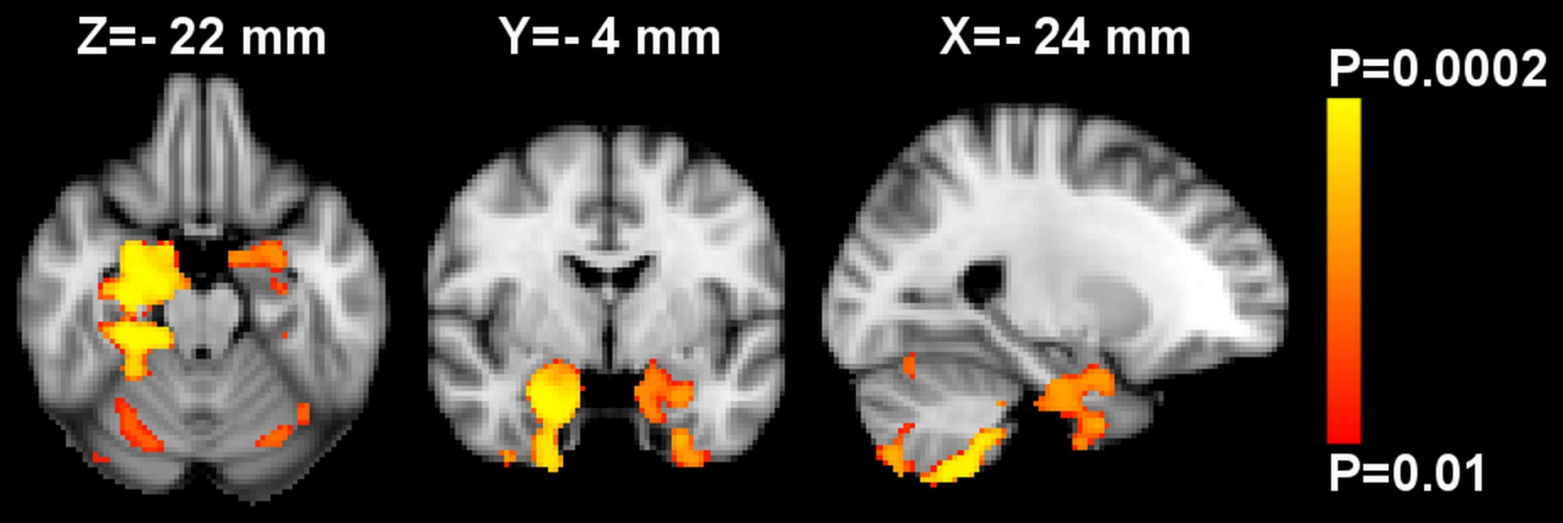
Whole-brain voxel-based morphometry analysis of gray matter density (GMD). Red-yellow shows voxels demonstrating significant inverse association between GMD and BMI z-score at T4 (i.e. at the day of MRI) after controlling for age and sex. Color bar represents P-values corrected for multiple comparisons using “threshold-free cluster enhancement”. The map of P-values was thresholded using corrected P≤0.01. The background image is the MNI152 standard space T_1_ template. X-, Y- and Z-values indicate the MNI slice coordinates in millimeter. Images are shown in radiological convention.

After investigating further, it turned out that GMD was inversely associated with zBMI bilaterally in the amygdala (P≤0.01, Fig 2) and accumbens (P≤0.05). These effects were significant when performing whole-brain analysis adjusted for age and sex. There were no significant interactive effects of age*sex, zBMI*age or zBMI*sex on GMD.

Replacing age by the Tanner scale based puberty stage did not change the above significance pattern, except that the inverse association of GMD and zBMI in the left accumbens didn’t survive the TFCE correction. There were no significant interactive effects of puberty*sex, zBMI*puberty or zBMI*sex on GMD.

## Discussion

### MR volumetry

Our results revealed that increased zBMI in children/adolescents is associated with increased left and right amygdalar volumes and enlarged accumbens.

The finding of increased amygdala volume related to overweight/obesity is consistent with our earlier MR volumetry study in young adults [26], as well as with a study in a large cohort of elderly subjects [41]. While a recent VBM study in adolescents reported a seemingly contradictory result: smaller GMM in the bilateral amygdala of obese participants [21]. However, in the temporal lobe of youths an opposite directional change in GMM (also measured during VBM analysis) and volume is not without example [25]; see our discussion on VBM results below.

The primary regions regulating food intake are the hypothalamus and the nucleus tractus solitarii mostly based on actual caloric and nutritional requirements. Other brain regions govern hedonic food intake, such as limbic areas including the amygdala, nucleus accumbens and hippocampus [42–44].

The amygdala has long been accepted to be involved in emotional processing, and especially in Pavlovian learning processes that impact upon appetitive and aversive behavior [45]. Eating provides the energy for general metabolism, and as such, the homeostatic control of energy intake is evident. However, feeding is not solely driven by the homeostatic needs, other higher order cognitive processes also affect appetite. Craving for palatable (usually sweet or high energy) food is a well-known phenomenon. Taste can significantly alter the behavioral response to food stimuli and promote overeating which is partly mediated by the amygdala having a well-described sensory input from chemical receptors within the oral cavity via brainstem and thalamus [46]. Modern neuroimaging studies also showed the primal role of amygdala in the processing of food related information [47] and reported higher amygdalar activation for food stimuli in obese individuals compared to lean subjects [48]. Mehta et al. showed that amygdala activation in response to food cues depends on satiety state and concluded that the activation of this region (along with the dorsal striatum and the accumbens) may alter the motivation to consume high calorie food (i.e. driving food choice) [47]. Amygdala has another main function that also affects food related information processing; amygdala facilitates attention to salient information [49]. One hypothesis behind this phenomenon stated that salient information is detected directly by the amygdala and its cortical projections facilitate attention and perception related to that stimulus [50, 51]. A recent fMRI study revealed that the salience encoding function of amygdala is non-linearly affected by BMI and concluded that it contributes to differences in neural craving regulation [52]. These complex functions of the amygdala related to energy intake regulation imply that a change in the morphology of this structure (e.g. enlargement) is linked to the impairment of energy homeostasis.

In support of our finding in the accumbens, a recent genetic study in children (9–12 years) found that children at higher risk for obesity exhibited stronger responses in the accumbens to food commercials and demonstrated larger accumbens volume [53]. However, another study in older adults (70–82 years) observed that fat mass and obesity-associated gene (FTO) risk allele was associated with smaller accumbens [54], warranting caution in interpretation. Using volumetry in middle-aged adults, obesity was found to be positively associated with the combined volume of left and right nucleus accumbens, which couldn’t be generalized to other dependencies (preserved accumbens volume was observed in subjects with alcohol dependence) [55]. Investigating healthy women, left accumbens was found to be significantly greater in the high BMI group compared with the lean group [56]. In adults, VBM-derived GMM in the accumbens was also found to be positively correlated with measures of obesity [57]. However, based on our results, VBM and volumetry should not be paralleled (at least in youths). To our knowledge this is the first report to show a positive association between overweight/obesity and accumbens volume in children/adolescents. Thus, our result points to the importance of the accumbens in overweight and obese children and adolescents.

Nucleus accumbens is part of the mesocorticolimbic pathway (the central component of the reward system). The main afferent information is received from the ventral tegmental area and the prefrontal cortex; these three interconnected regions form a circuit [58, 59]. Several studies demonstrated the central role of the mesocorticolimbic pathway. Dopamine, which is produced in the midbrain and stimulates the limbic areas such as the accumbens, is considered as a major nonhomeostatic influence over food intake [60]. Elevated dopamine levels were measured in rats within the nucleus accumbens following exposure to food [61], sweets [62], self-administered drugs [63], and sex [64]. Human imaging studies also confirmed these findings, showing prominent activation of the striatum in response to these stimuli [65–67] and additionally to money [68].

Due to conditioning over some time, neutral stimuli (cues of food or drugs), that are linked to the unconditioned stimuli, acquire the ability to increase dopamine in striatum (including the nucleus accumbens). This leads to the anticipation of the reward and consequently, forms a strong motivation to perform and sustain the newly learned behavior [69]. As a result of the above discussed findings, the homeostatic control over food consumption/energy intake becomes secondary to reward driven non-homeostatic regulation [70]. Animal studies examined the relation between nucleus accumbens and obesity even further; it was shown that altered dopamine and opioid signaling in the accumbens may contribute to the development of obesity, as it leads to maladaptive behaviors, such as excessive eating [71]. Later studies reported morphological alterations (modified dendritic spine density) within the nucleus accumbens as a response to operant food seeking behavior for highly palatable food [72]. The same group reported that prolonged exposure to high-calorie diet led to overeating and consequent overweight, and to a change in the functionality of the accumbens presenting structural plasticity modifications. These modifications were not only characterized by dendritic spine density, but increased expression of neuroinflammatory factors and activated microglia [73].

These findings suggest that the modified activation (e.g over-activation) of these structures (amygdala and accumbens) results in the override of homeostatic signals with reward-related information causing altered regulation of food intake that promotes the chronic positive energy balance leading to and/or maintaining obesity [70, 74].

### Voxel-based morphometry

Our region of interest VBM analysis indicated a significant inverse relationship between obesity and GMM bilaterally in the amygdala. By extracting mean GMM and mean GMD (i.e. the unmodulated version of GMM) from the significant left and right amygdala regions, partial correlation analysis showed that mean GMM was no longer associated with zBMI after controlling for mean GMD, suggesting that the association was mediated by GMD. Performing whole-brain VBM analysis for GMD, it turned out that GMD showed a significant inverse relationship with zBMI bilaterally in the amygdala and accumbens. In line with our finding, a recent VBM study reported that obese adolescents had reduced GMM in the amygdala bilaterally [21]. However, while they discussed their finding as reduced GM volume, our findings suggest that reduced GMM in obesity is rather related to reduced GMD and by measuring real volumes (in mm^3^) using volumetry, an obesity related volume increase can be detected.

It is not unusual that volume and GMD change in opposite directions in brains without severe neuronal loss. A recent study examining age-related effects from childhood to young adulthood (age range: 8–23 years) found that GMD and volume changed in different directions as a function of age–volume decreases, while density increases with age–[25]. In our study, the contradictory result could not be a function of age as we did not observe correlation between zBMI and age or interaction effects of age*zBMI and we adjusted for age in our analyses. However, agreeing with that study we also suggest that GMD and volume must be considered distinct and complementary. Others also suggested that GMM is sensitive to factors beyond brain volume (i.e. T1 signal alteration) [75], which may result in discordant changes between GMM and volume.

Although we found that GMD is sensitive to childhood obesity, its neurobiological basis is unclear. Actually GMD is a unitless scalar measure (i.e. gray matter probability) which should not be confused with cell packing density measured cytoarchitectonically [76]. While one of the most common interpretations of reduction in GMD is neuronal loss [77]–which is likely to be the case in Alzheimer’s disease or other neurodegenerative diseases–this interpretation may be problematic in healthy subjects or in diseases without extensive neuronal loss [78]. The few combined histological and MRI studies found no correlations between GM probability values and histological measures in macroscopically normal appearing GM [77, 79]. However, these studies examined only a limited number of histological measures in the cortical crown of the middle temporal gyrus, which cannot be generalized to other cortical or subcortical regions. There are myriad conditions (e.g. myelin, iron and water content or cellular morphology differences) that theoretically can affect T_1_–weighted MRI signal and thereby the calculated GMD. However, without histological proof, the actual cause is highly speculative and such speculation is beyond the scope of this discussion. Nevertheless, the co-localization of volume and density changes suggests that the associations we found might be of importance.

### Methodological considerations

In volumetry, we used the Benjamini and Hochberg procedure with q = 0.1 to correct for multiple comparisons. Although it can be considered a liberal correction, it should be also taken into account that left and right hemispheric brain structures (e.g. left hippocampus vs. right hippocampus) and zBMI values at the two timepoints (i.e. zBMI T4 vs. zBMI T3) are highly correlated and cannot be assumed as independent observations. To avoid data-fishing we restricted our investigation to only a small number of predefined reward-related brain regions having good reasons to believe that they could be involved in obesity; similarly to our previous study [26]. Because both amygdala and accumbens were related with zBMI in both hemispheres, it seems unlikely that our data are driven by chance alone. Moreover, even after the most conservative correction (Bonferroni correction: 10 regions*2 timepoints) the right accumbens at T4 and the left amygdala at T3 would remain significantly associated with zBMI.

There are other reward-related brain regions, which cannot be examined with the applied volumetric method. One of them is the orbitofrontal cortex, which was often missegmented in our young population, while the other is hypothalamus for which there is no automatic label in Freesurfer.

Due to the cross-sectional design and the possible bidirectional relationship between brain volume and obesity the cause and effect could not be discriminated in this study, but based on the limited ages of our subjects, it can be speculated that either increased brain volume is a cause of overweight/obesity or a short duration of obesity may already induce significant morphologic changes in the developing brain. So far only two VBM studies exist in children/adolescents addressing the obesity-related brain changes longitudinally, which indicated that BMI and body fat gain resulted decreased regional GMM in the right posterior medial temporal lobe and the right putamen, respectively [80, 81], but our knowledge is still quite limited. Longitudinal volumetry studies and measurements at more than two time points are also needed to address this question adequately.

The study is limited by the modest sample size and number of subjects with excess weight. Thirteen subjects (25%) had excess body weight at T4 based on categorization by Cole et al.[40] and 15 subjects (29%) had a zBMI value bigger than 1 (also commonly used as cutoff for excess weight). This is partly due to our intention to minimize the chance of obtaining spurious results by rigorous selection of subjects with the highest image quality (more than one third of the subjects had been excluded due to signs of motion or segmentation problem). This exclusion rate is not unique to our study, it is comparable to some previous MRI studies investigating children [82, 83], while others reported higher success rates [84]. To overcome this, zBMI was handled as a continuous measurein all of our analyses. Comparison to other studies must be performed in light of the above. Future studies with larger sample size are needed to replicate and further investigate the associations between obesity and brain structure in children/adolescents.

Using the prevalence values calculated in our earlier study according to the three most widely accepted definitions of the pediatric metabolic syndrome (International Diabetes Federation consensus definition in children and adolescents; National Cholesterol Education Program definition adapted to adolescents by Cook et al.; definition of Viner et al.) [85], it is unlikely that even a single subject with metabolic syndrome (MetS) is present among our 51 subjects. However, in the present study no blood samples were collected, thus we can’t rule out the incidental presence of MetS subjects. Yau et al. suggested smaller hippocampal volume in adolescents with MetS [86], while the same group didn’t find hippocampal volume changes in uncomplicated obese adolescents (without MetS) [87], which is in line with our present findings. Unfortunately no amygdala or accumbens were investigated in these studies, precluding further in-depth comparison to our results.

Nevertheless, our study has significant strengths, including the utilization of both volumetry and VBM methods, and the evaluation of both past and current obesity measures (i.e. T3 and T4 respectively) on GM volume.

## Conclusions

In conclusion, the present study shows that overweight/obesity related brain differences may already be present in children/adolescents. Our results suggest that a higher degree of obesity in children/adolescents is associated with greater volumes of amygdala and accumbens; both regions involved in motivated behavior related to food intake regulation. Future longitudinal studies should clarify the causality and whether these early brain differences are reversible or not. Somewhat unexpectedly, the GMD of both amygdala and accumbens was negatively correlated with obesity, although currently it is not clear whether this pattern is a consequence of histological alterations or the methodological limitations specific to VBM analysis. The disparity found between VBM-derived measures and volume in the context of obesity in children/adolescents may inspire new volumetry studies and warrants cautious interpretation of VBM studies in obesity.

## Supporting information

S1 Fig Mean gray matter mass is no further associated with zBMI after controlling for mean gray matter density. Part (a) and (c) show significant negative correlations (P<0.05, two-tailed Pearson correlation) between BMI z-score (zBMI) at T4 and both left and right amygdalar gray matter mass (GMM) averaged over the voxels where voxel-based morphometry showed significant association between GMM and zBMI. Partial regression plots demonstrate that mean GMM was no further associated (two-tailed P>0.2) with zBMI after controlling for mean gray matter density (GMD); b and d respectively for the left and right amygdala. Linear correlation coefficients (r) are also presented.
(TIF)
